# Differential influences of (±) anatoxin-a on photolocomotor behavior and gene transcription in larval zebrafish and fathead minnows

**DOI:** 10.1186/s12302-021-00479-x

**Published:** 2021-03-30

**Authors:** Lea M. Lovin, Sujin Kim, Raegyn B. Taylor, Kendall R. Scarlett, Laura M. Langan, C. Kevin Chambliss, Saurabh Chatterjee, J. Thad Scott, Bryan W. Brooks

**Affiliations:** 1 Department of Environmental Science, Baylor University, Waco, TX 76798, USA.; 2 Department of Chemistry, Baylor University, Waco, TX 76798, USA.; 3 Department of Environmental Health Sciences, University of South Carolina, Columbia, SC 29208, USA.; 4 Department of Biology, Baylor University, Waco, TX 76798, USA.

**Keywords:** Harmful algal blooms, Cyanobacteria, Natural toxins, Anatoxin-a, Water quality, Comparative toxicology

## Abstract

**Background:**

Though anatoxin-a (antx-a) is a globally important cyanobacterial neurotoxin in inland waters, information on sublethal toxicological responses of aquatic organisms is limited. We examined influences of (±) antx-a (11–3490 μg/L) on photolocomotor behavioral responses and gene transcription associated with neurotoxicity, oxidative stress and hepatotoxicity, in two of the most common alternative vertebrate and fish models, *Danio rerio* (zebrafish) and *Pimephales promelas* (fathead minnow). We selected environmentally relevant treatment levels from probabilistic exposure distributions, employed standardized experimental designs, and analytically verified treatment levels using isotope-dilution liquid chromatography tandem mass spectrometry. Caffeine was examined as a positive control.

**Results:**

Caffeine influences on fish behavior responses were similar to previous studies. Following exposure to (±) antx-a, no significant photolocomotor effects were observed during light and dark transitions for either species. Though zebrafish behavioral responses profiles were not significantly affected by (±) antx-a at the environmentally relevant treatment levels examined, fathead minnow stimulatory behavior was significantly reduced in the 145–1960 μg/L treatment levels. In addition, no significant changes in transcription of target genes were observed in zebrafish; however, *elavl3* and *sod1* were upregulated and *gst* and *cyp3a126* were significantly downregulated in fathead minnows.

**Conclusion:**

We observed differential influences of (±) antx-a on swimming behavior and gene transcription in two of the most common larval fish models employed for prospective and retrospective assessment of environmental contaminants and water quality conditions. Sublethal responses of fathead minnows were consistently more sensitive than zebrafish to this neurotoxin at the environmentally relevant concentrations examined. Future studies are needed to understand such interspecies differences, the enantioselective toxicity of this compound, molecular initiation events within adverse outcome pathways, and subsequent individual and population risks for this emerging water quality threat.

## Background

Though cyanobacteria are important primary producers in freshwater and marine ecosystems, large-scale blooms of harmful species present risks to human health and ecosystems when elevated levels of toxins are produced. Site-specific cyanobacterial and other harmful algal blooms in inland waters can cause more pronounced impacts on environmental quality than many conventional chemical contamination events [1]. Toxins produced during cyanobacterial blooms vary widely with numerous compounds classified by mechanism of action and structure [2], along with other substances for which environmental fate and toxicological profiles are largely unknown. Reported responses following exposure include neurotoxicity, hepatotoxicity, dermatotoxicity, immunotoxicity and other adverse outcomes in diverse organisms [3]. Cyanotoxins levels in aquatic systems are elevated by higher cell density when blooms occur, but toxins biosynthesis is influenced by genetic factors and environmental conditions such as temperature [4, 5], light [6, 7], and nutrient levels and stoichiometry [8-10]. Understanding aquatic conditions that lead to production and release of toxins and subsequent consequences is key to protecting ecosystems and public health, especially since bloom magnitude, frequency and duration appear to be increasing with climate change [11-13].

Some of the most common neurotoxic cyanobacterial toxins are anatoxins, which have been identified in over 30 countries during blooms of *Aphanizomenon, Dolichospermum* (prev. *Anabaena*), *Microcystis, Nostoc, Oscillatoria, Planktothrix, Phormidum, Raphidiopsis* and other pelagic and benthic cyanobacterial genera [14]. The most frequently reported form of anatoxin is anatoxin-a (antx-a), which can accumulate in fish and other aquatic organisms [15-18]. Antx-a is a chiral, bicyclic amine that binds irreversibly to nicotinic acetylcholine receptors with a higher affinity than acetylcholine and is not hydrolyzed by acetylcholinesterase [19-22], though its mechanism of action is not fully elucidated. Studies have implicated antx-a in the death of fish, dogs, bats, livestock, and birds [23-26]. However, this compound has received much less study than other cyanobacterial toxins such as microcystins and saxitoxins [2]. Robust toxicity studies of antx-a with aquatic organisms are limited, with the majority of previous efforts failing to analytically verify treatment levels or employ standardized experimental designs [14]. Importantly, toxicity assays using the racemic mixture, (±) antx-a, are widely reported in literature, although only one enantiomer, (+) antx-a, has been described in aquatic systems [15], and is more potent in frogs and rodent models [20, 27-29]. For example, LD_50_ values for mice administered intravenously were observed to be 386 μg/kg for (+) antx-a, compared to 913 μg/kg for (±) antx-a, and no deaths were observed in mice up to 73 mg/kg for (−) antx-a [27].

Sublethal toxicity of antx-a is poorly understood, particularly in aquatic organisms, which includes increasingly common alternative vertebrate models for biomedical applications [14]. Previous aquatic toxicology studies with antx-a have not consistently stated the purity of toxin under investigation or which enantiomers were studied and a number have examined organismal responses following exposure to cultures that may differentially produce antx-a and other bioactive molecules [14]. For example, exposure of pure (±) antx-a at 80–640 μg/L only reduced standard length in carp, while exposure to extracts of *Anabaena* sp. (ANA 37) containing (+) antx-a at 83–666 μg/L were highly toxic [30]. In zebrafish, 400 μg/L of an undefined antx-a enantiomeric mixture temporarily altered heart rate in a developmental stage-dependent fashion, with heart rate decreasing 9% at 55 h and increasing 12% at 80 h [31]. Further, when rainbow trout were exposed to an unspecified enantiomeric mixture of antx-a, immediate abnormal behavioral effects (irregular/erratic swimming, jaw spasms, swimming near surface with mouth in air, difficulty maintaining equilibrium) were noted, followed by fish recovery by 3 h [32]. Thus, an understanding of the aquatic toxicology of antx-a has remained elusive.

In the present study, we investigated sublethal toxicity of (±) antx-a influences in embryonic and larval zebrafish and fathead minnow models. We explored whether and the extent to which behavioral and gene transcriptional endpoints are affected by (±) antx-a in these common fish models, following exposure to experimental treatment levels selected from centiles of a probabilistic exposure distribution of antx-a in surface waters [14].

## Methods

### Fish culture

Tropical 5D wild-type zebrafish (*Danio rerio*) were maintained at Baylor University (Waco, Texas, USA) following standard culturing conditions described previously [33-35]. Zebrafish were housed in a Z-Mod recirculating system (Marine Biotech Systems, Beverly, Massachusetts, USA) at a density of < 4 fish per liter. Temperature was held at 28 ± 1°C, pH at 7.0 ± 0.1, and salinity at 260 ppm (Instant Ocean). Fish were fed twice daily with artemia (Artemia sp. nauplii; Pentair AES, Apopka, Florida, USA) and once daily with flake food (Pentair AES, Apopka, Florida, USA) under a 16-h:8-h light:dark photoperiod. Fathead minnow (*Pimephales promelas*) larvae were acquired < 48 h post-hatch (Environmental Consulting and Testing, Superior WI, USA). Culture conditions were maintained at 25° C ± 1 °C and pH varied from 7.8 to 8.1. All experimental procedures and fish-culturing protocols followed Institutional Animal Care and Use Committee protocols approved at Baylor University.

### Experimental design

To ensure comparability of this study to other efforts, standardized experimental methods from the Organisation for Economic Co-operation and Development (OECD) guidelines for toxicity testing with zebrafish [36] and US Environmental Protection Agency (EPA) for fathead minnows [37] were modified for use in studying specific behavioral [34, 35] and gene transcriptional endpoints [33]. Solutions of (±) antx-a (> 98%; CAS 64285-06-9; Abcam, Cambridge, UK) and caffeine (> 95%; CAS 58-08-2; Sigma-Aldrich, St. Louis, Missouri, USA), which was used as a behavioral positive control [35], were prepared in reconstituted hard water (RHW) [38]. Since antx-a is an ionizable weak base, solutions were titrated to pH 7.5 for ionization state consistency among experiments [37, 39]. Common water quality parameters (dissolved oxygen, temperature, conductivity, alkalinity, and hardness) of the RHW used for all experiments were routinely measured during experimentation.

Zebrafish embryos were exposed at 4–6 h post-fertilization (hpf) and placed in 100-mL glass beakers containing 52 mL of solution (4 replicate experimental units: 26 embryos in each, 2-mL solution per embryo) in an incubator at 28 °C. Embryos were from the same batch and the experiment was performed at the same time, except for the 3000 μg/L treatment level, which was conducted during a subsequent experiment. Fathead minnow larvae < 48 h post-hatch were placed in 500-mL glass beakers containing 300 mL of exposure water (4 replicate experimental units: 15 larvae in each, 20 mL per larvae) at the same time in an incubator at 25 °C. Incubators were maintained on backup power with the photoperiod for both species 16-h:8-h light:dark. Nominal treatment levels were determined based on environmental exposure distributions with the highest concentration (1500 μg/L) corresponding with the 97th centile of reservoir occurrence data [14]. Both species were exposed at nominal concentrations of 10, 100, 500, 1000, and 1500 μg/L. In a follow-up experiment using zebrafish, (±) antx-a was increased to examine an additional 3000 μg/L treatment level. The higher concentration experiment was completed after the lower treatment levels were analyzed to inform future toxicology studies. Caffeine was selected as a positive control due to activity as a cholinergic agonist [40]. Caffeine treatments (412 μg/L for zebrafish, 56,380 μg/L in fathead minnow) were based on levels that elicited a significant behavioral response in prior research [35]. For 96 h of exposure, water changes occurred daily for zebrafish and at 48 h for fathead minnows. Fish were checked daily for mortality and developmental abnormalities, with dead fish removed from experimental units. Following the experiment, 6 zebrafish larvae (4 replicates, ~ 100–102 hpf) from each treatment level were placed individually into 48-well plates with 1 mL of exposure water [35]. For fathead minnow, 4 larvae (3 behavioral replicates, ~ 144 hph) were placed into 24-well plates in 2 mL of exposure water due to their larger size [35]. Only larvae with no clear developmental malformations (bent spines, edemas, etc.) were employed for behavioral assays [41]. Organisms allowed to acclimatize in the incubator prior to being loaded in the behavioral system with consistent acclimation times among the plates [38].

### Photolocomotor behavioral analyses

Following previous methods [34, 35, 42], larval photolocomotor activity was recorded using automated tracking software and associated platform (Zebrabox, ViewPoint, Lyon, France). Behavioral analyses were initiated from 12:00 to 15:00 to decrease time of day-related changes in behavior [42, 43]. The ViewPoint system was set in tracking mode and behavioral recordings occurred over 50 min. Recording started with a 10-min dark acclimation followed by a 40-min observation period consisting of two altering 10-min light/dark cycles. Distance swam, changes in number of movements (counts), and duration of movements across three speed thresholds: bursting (> 20 mm/s), cruising (5–20 mm/s), and freezing (< 5 mm/s) were recorded at 1-min intervals. To measure larval swimming responses to a sudden change in light condition, a photomotor response was observed following methods previously used [44] with slight modifications [34]. Photomotor response for each photoperiod transition (2 light and 2 dark periods) was calculated as the change in mean distance traveled (in mm) between the last minute of an initial photoperiod and the first minute of the following period. Photomotor responses were observed across each speed threshold (bursting, cruising, and freezing) in addition to total distance.

### Gene transcription

Total RNA and protein were simultaneously extracted from 21 to 24 zebrafish larvae per beaker with 4 replicates (*n* = 4) and 13–15 fathead minnow larvae per beaker with 4 replicates (*n* = 4) after the 96-h exposure period using an AllPrep RNA/Protein Kit (Qiagen, Hilden, Germany) following manufacturer’s instructions with minor modifications. Fish from the behavioral experiment and the remaining fish in the experimental units were used for analysis. Specifically, following homogenization, samples were incubated for 5 min at 37 °C with the extraction proceeding according to instructions thereafter. While extracted protein was kept at −80 °C for future studies, quality of total RNA was evaluated using a NanoDrop One Microvolume UV–Vis Spectrophotometer (Thermo Fisher Scientific, Waltham, Massachusetts, USA). Total RNA with an A_260/280_ > 1.8 was cDNA converted with ~ 1000 ng for zebrafish and 500 ng for fathead minnow for experiment 1 (0–1.5 mg) and ~ 500 ng converted for experiment 2 (0–3 mg) for zebrafish using TaqMan Reverse Transcription Reagents (Invitrogen, Carlsbad, CA, USA). Primers sets were designed using the National Center for Biotechnology Information (NCBI) primer blast tool or taken from the literature (Additional file 1: Table S1). The qualities of the PCR products were confirmed on a 2% agarose gel with SYBR safe staining (Invitrogen).

Two-step RT-qPCR was done with Power SYBR Green PCR Master Mix (Applied Biosystems, Foster City, CA, USA). The 20-μL reaction mix consisted of 10 μL of the PCR master mix, 0.6 μL of each 10 μM PCR primer (IDT, Coralville, IA, USA), 7.8 μL of nanopure water, and 1 μL template cDNA (1: ~ 20 ratio used). RT-qPCR was carried out on a QuantStudio 6 Flex Real-Time PCR system (Thermo Fisher Scientific). The thermal cycle profile was: preincubation at 95 °C for 10 s and 60 °C for 1 min with melting curve analysis. Transcript levels were normalized to housekeeping genes using the 2^−ΔΔ*C*^_T_ method [45]. Based on initial geNorm analysis of 3 potential housekeeping genes (data not shown), elongation factor 1 alpha (*elfα*) for zebrafish and 18s ribosomal RNA (*18s rRNA*) for fathead minnows were used as housekeeping genes.

### Analytical measures

Experimental treatment levels of (±) antx-a were analytically verified using a previously published isotope-dilution liquid chromatography tandem mass spectrometry (LC–MS/MS) method [46]. Briefly, samples were collected and diluted accordingly in 10:90 (v/v) nanopure water:acetonitrile buffered with 5 mM ammonium formate and 3.6 mM formic acid (pH 3.7). Diluted sample (990 μL) was added to a 2-mL autosampler vial and spiked with 10 μL of antx-a-13C4 (1 μg/mL). Quantification was completed using previously described method parameters on a 1260 High-Performance Liquid Chromatography system equipped with a Poroshell HILIC-Z column (2.1 × 150 mm, 2.7 μm, 120 Å) and G6420 triple quadrupole mass spectrometer (Agilent, Santa Clara, CA) [46].

### Statistical analyses

Statistical analyses for survival, behavior, and RT-qPCR data were carried out in SPSS Statistics 27 (IBM, Armonk, NY, USA). Data were examined for normality by Shapiro–Wilk’s test and for homogeneity by Levene’s test. Behavioral analyses were performed for each treatment with 6 zebrafish larvae (4 replicates), and 4 fathead minnow larvae (3 replicates), which is consistent with our previous work with these species [34, 35, 42]. Survival of the negative control to the exposure treatments was compared with a Fisher’s exact test (*α* = 0.05). Independent samples *t* tests for the caffeine positive control vs the negative control, and one-way analysis of variance (ANOVA) tests for antx-a treatment levels and the negative control were performed for the behavioral data (*α* = 0.10), and transcription was analyzed using the 2^−ΔΔ*C*^_T_ method [45] for the RT-qPCR data (*α* = 0.05), after parametric testing criteria was met. Dunnett’s post hoc tests were performed to identify potential differences among treatment levels. Non-parametric Kruskal–Wallis tests and Mann–Whitney U tests were performed when data did not pass ANOVA testing criteria even after log transformation.

## Results

### Analytical verification of experimental treatment levels

Measured levels of (±) antx-a were 11, 118, 671, 1310, 1950, and 3490 μg/L for the zebrafish studies, and 12, 145, 682, 1450, and 1960 μg/L for the fathead minnow experiment. Both were slightly higher than nominal concentrations (14.0–44.7%) with no (±) antx-a detected in the controls. Due to differences between the analytically verified and nominal concentrations, only measured concentrations are used for subsequent results and discussion.

### Survival and developmental abnormalities

Mortality in negative control fish was < 10% at 96 h and no (±) antx-a or caffeine treatment had significantly different survivability using Fisher’s exact test (α = 0.05). While there was low mortality for all treatment levels, almost all zebrafish deaths occurred within 24 h, while fathead minnow mortalities occurred mostly by 96 h. There were few developmental abnormalities in both species (~ 1%), which mainly consisted of bent spines.

### Behavior of negative and positive controls

**Fig. 1:**
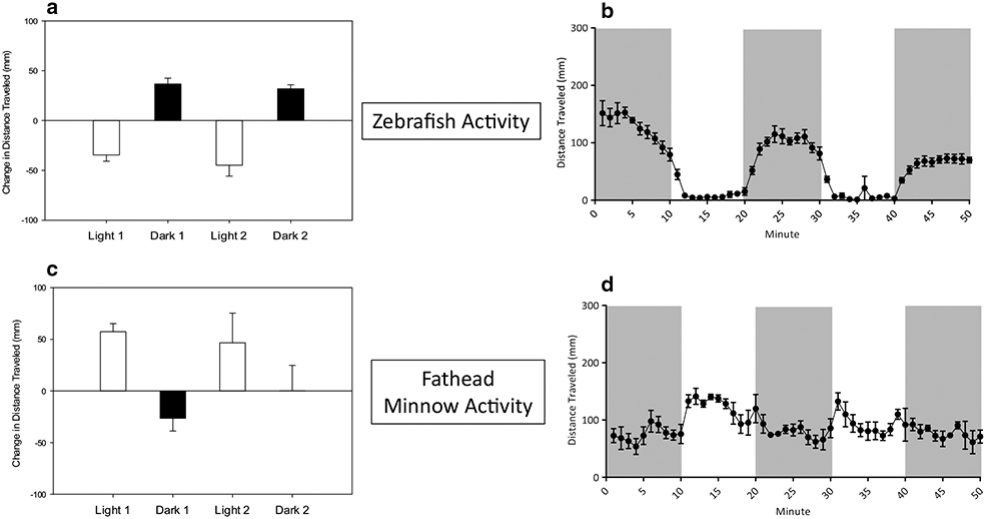
Photomotor response and total locomotor behavior of unexposed zebrafish (**a**, **b**) and fathead minnows (**c**, **d**). **a** and **c** show photomotor response measured as the change in mean (± SE) total distance traveled between the last minute of the prior photoperiod and the first minute of the following period. **b**, **d** show mean (± SE) distance swam at each minute interval. Dark gray bars represent activity in the dark and the white bars represent activity in the light. A total of 24 zebrafish (4 replicates each with 6 larvae) and 12 fathead minnows (3 replicates with 4 larvae) were used for baseline behavioral observation

**Fig. 2:**
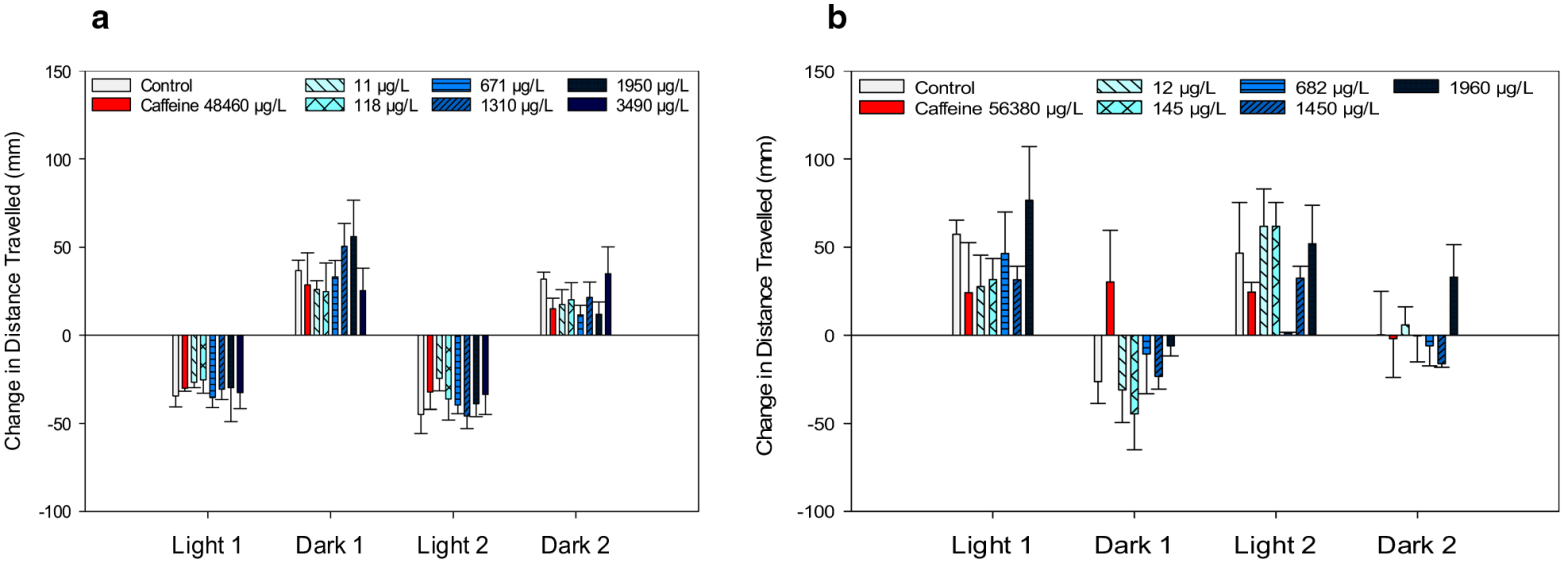
Photomotor response of zebrafish (**a**) and fathead minnow (**b**) exposed to caffeine or (±) anatoxin-a measured as the change in mean (± SE) total distance traveled between the last minute of the prior photoperiod and the first minute of the following period. A total of 24 zebrafish (4 replicates each with 6 larvae) and 12 fathead minnows (3 replicates with 4 larvae) were used for each treatment level

In the negative control, photolocomotor activity of larval zebrafish and fathead minnows were similar to previous reports from our laboratory [34, 35, 42, 47]. For example, zebrafish increased movement in dark and decreased movement in light conditions (Fig. 1a), and fathead minnows increased movement in light and decreased movement in dark conditions (Fig. 1c). Activity of negative control fish at each minute of the experiment indicated that zebrafish (Fig. 1b) changed movement patterns at each light cue and stayed at relatively steady plateaus of movement during each period represented by gray (dark activity) and white (light activity) blocks. Fathead minnow activity (Fig. 1d) included more variable behavior during each period with changes in movement pattern occurring without a concurrent light cue. Caffeine exposure of 412 μg/L to zebrafish and 56,380 μg/L to the fathead minnow significantly lowered (*p* < 0.05) total count, cruising distance, cruising count, and freezing distance in zebrafish, and significantly (*p* < 0.1) decreased bursting distance, count, and duration in dark conditions and total count, cruising distance, count, and duration, and freezing count of fathead minnows. In both species, caffeine did not elicit significant (α = 0.1) photomotor changes between the light/dark period transitions (Fig. 2a, b).

### Behavioral responses to (±) antx-a

**Fig. 3:**
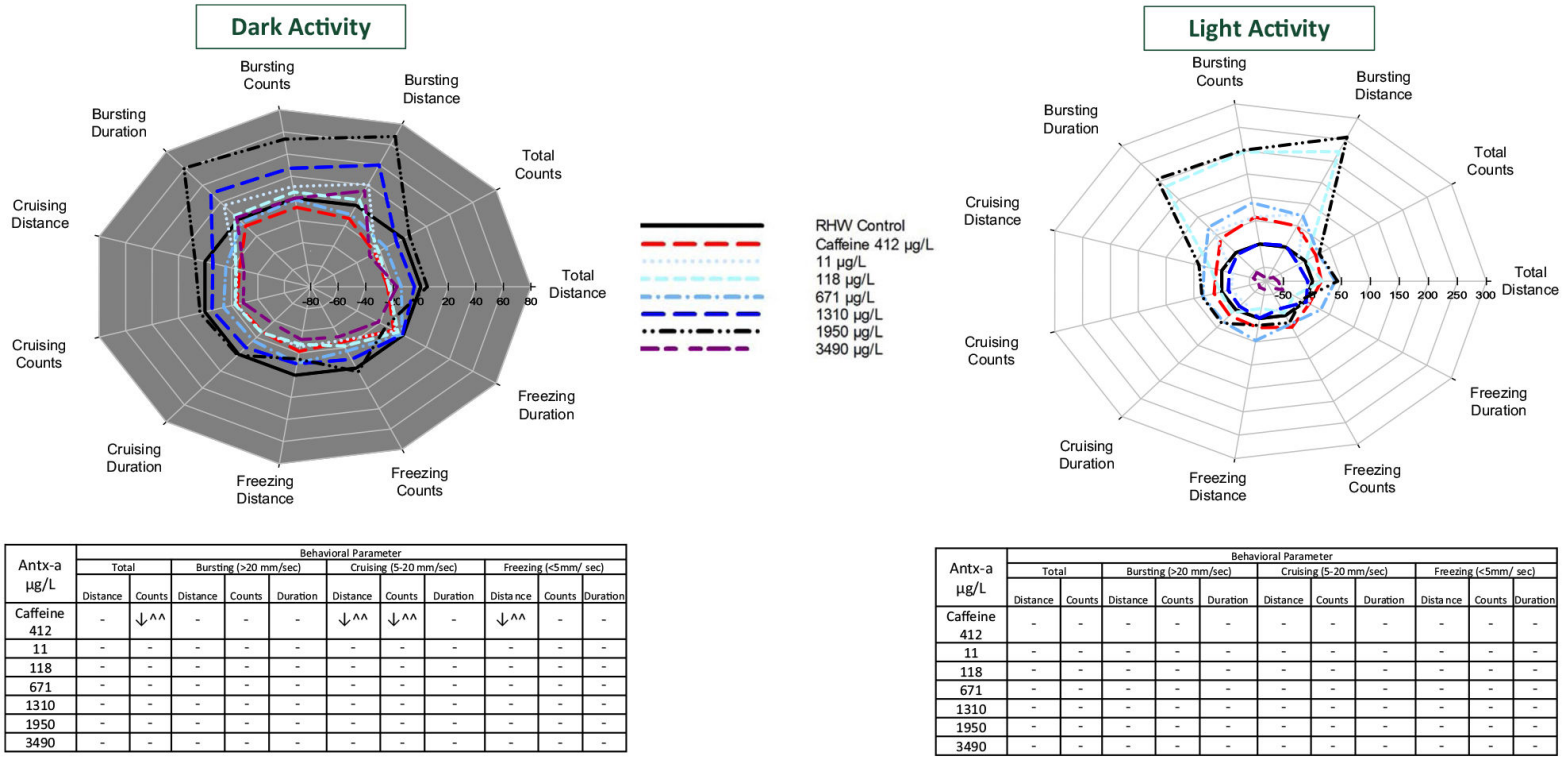
Behavioral response profiles of mean zebrafish swimming movement and speed in the dark (**a**) and in the light (**b**) each comprising 20 min (2 intervals of 10 min) after 96 h exposure to (±) anatoxin-a or caffeine. Behavioral parameters include swim distance, number of changes in movement (count), and swim duration in total and across 3 speeds, bursting (> 20 mm/s), cruising (5–20 mm/s), and freezing (< 5 mm/s). The tables below the graphs indicate a significant increase (↑) or decrease (↓) in activity compared to the negative control. In the (±) antx-a treatments, ANOVA and Dunnett’s post hoc were used to analyze treatment level responses compared to the negative control (*p < 0.10; **p < 0.05; ***p < 0.01). *T* tests were used to analyze caffeine influences compared to the negative control (^ *p* < 0.10; ^^ *p* < 0.05; ^^^ *p* < 0.01). A total of 24 zebrafish (4 replicates each with 6 larvae) were used for each treatment level

**Fig. 4:**
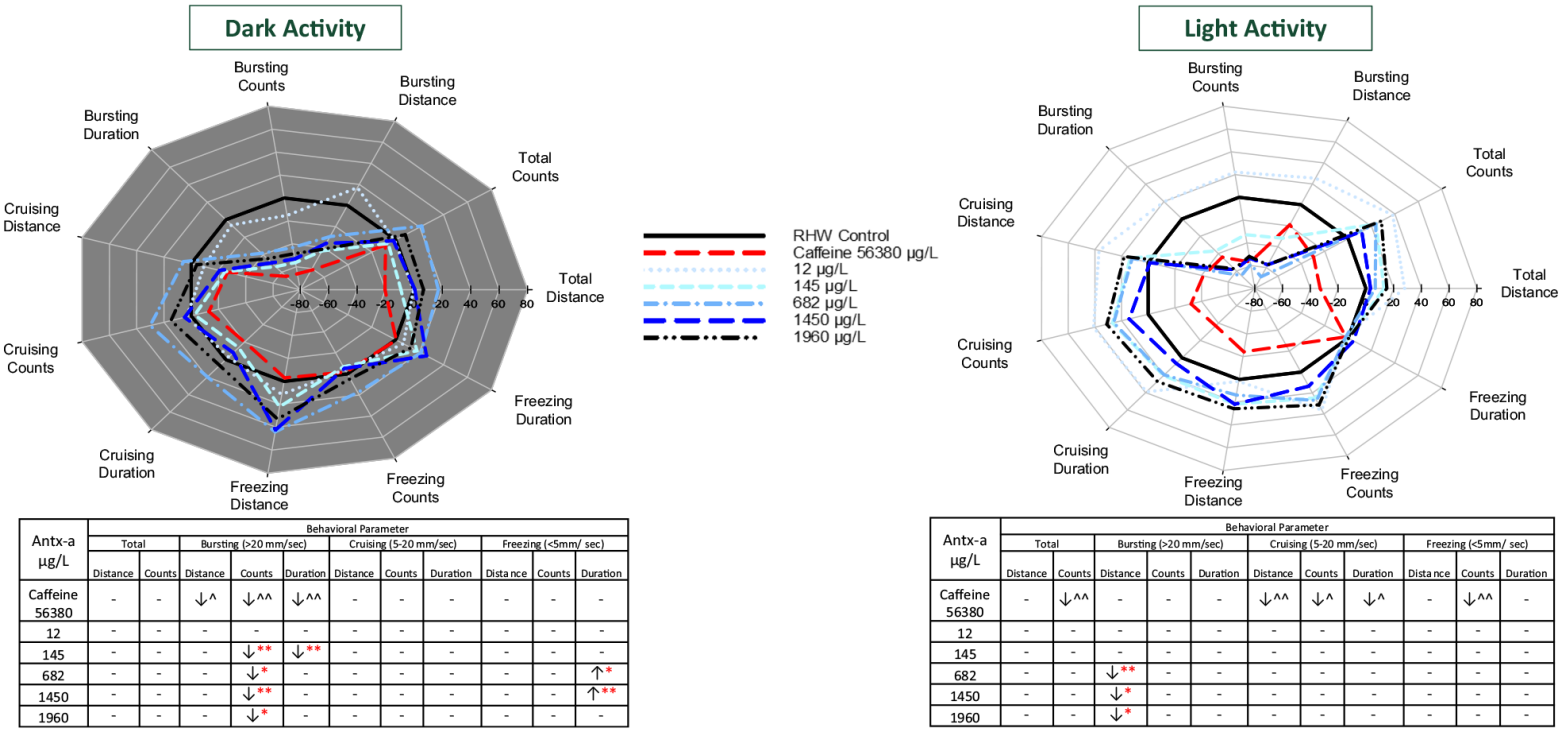
Behavioral response profiles of mean fathead minnow swimming movement and speed in the dark (**a**) and in the light (**b**) each comprising 20 min (2 intervals of 10 min) after 96-h exposure to (±) anatoxin-a or caffeine. Behavioral parameters include swim distance, number of changes in movement (count), and swim duration in total and across 3 speeds, bursting (> 20 mm/s), cruising (5–20 mm/s), and freezing (< 5 mm/s). The tables below the graphs indicate a significant increase (↑) or decrease (↓) in activity compared to the negative control. In the (±) antx-a treatments, ANOVA and Dunnett’s post hoc were used to analyze treatment level responses compared to the negative control (*p < 0.10; ** *p* < 0.05; ***p < 0.01), *T* tests were used to identify potential caffeine influences compared to the negative control (^ *p* < 0.10; ^^ *p* < 0.05; ^^^ *p* < 0.01). A total 12 fathead minnows (3 replicates with 4 larvae) were used for each treatment level

While exposed zebrafish showed consistent increased movement in transitions from light to dark and decreased movement in transitions from dark to light at similar levels to the negative control (Fig. 2a), fathead minnows had a more variable photomotor response (Fig. 2b), particularly in the transition to dark period 2. Zebrafish behavioral response profiles (Fig. 3) indicated stimulatory movement at the highest speed threshold (> 20 mm/s) for bursting distance, count, and duration during dark conditions, and for all endpoints in light conditions for the 11 through 1950 μg/L (±) antx-a treatment levels. These responses, though not statistically significant (*α* = 0.1), were more pronounced in the light period. We further examined a higher level of (±) antx-a at 3490 μg/L. Here again, zebrafish behaved similarly to the lower concentrations in the dark conditions, with slight stimulation at the 3 bursting endpoints. However, activity in the light tended to be lower for all variables, which was opposite of lower treatment levels though these responses were also not statistically significant. In contrast, fathead minnows showed opposite locomotor behavioral profiles from zebrafish. Bursting swim behavior was generally refractory in both the dark and light following (±) antx-a exposure. As displayed in Fig. 4, fathead minnow bursting count was significantly reduced by the higher treatment levels, including 145 (*p* < 0.05), 682 (*p* < 0.1), 1450 (*p* < 0.05), and 1960 (*p* < 0.1) μg/L, with cruising duration (*p* < 0.05) lowered in the 145 μg/L treatment. Light behavior showed a similar trend in refractory behavior for most treatments, though the lowest treatment level (12 μg/L) exhibited a slightly stimulatory locomotor response for most endpoints.

### Gene transcription responses (±) antx-a

**Fig. 5:**
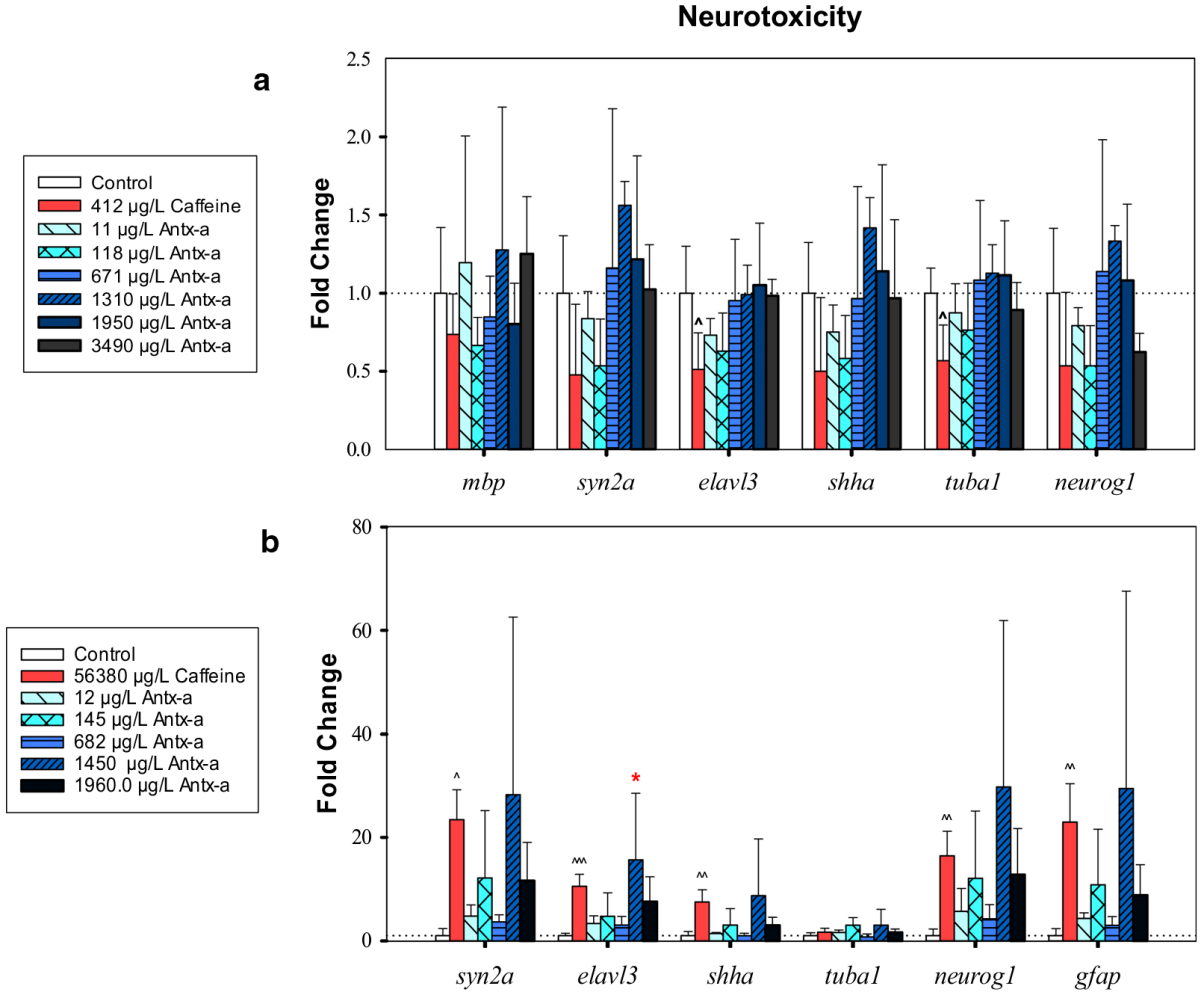
RT-qPCR neurotoxicity-related gene expression data for zebrafish (**a**) or fathead minnows (**b**) exposed for 96 h to (±) antx-a or caffeine compared to the negative control. Transcript levels were normalized to housekeeping gene, *elongation factor 1 alpha* in zebrafish and *18s ribosomal RNA* in fathead minnow, using the 2^−ΔΔ*C*^_T_ method. In the (±) antx-a treatments, ANOVA and Dunnett’s post hoc were used to analyze treatment level responses compared to the negative control (*p < 0.05; **p < 0.01; ***p < 0.001). *T* tests were used identify potential caffeine influences compared to the negative control ^ *p* < 0.05; ^^ *p* < 0.01; ^^^ *p* < 0.001, error bars (± SD). Zebrafish included 4 replicates with 21–24 larvae used for each treatment level. Fathead minnows included 4 replicates with 13–15 larvae used for each treatment level

**Fig. 6:**
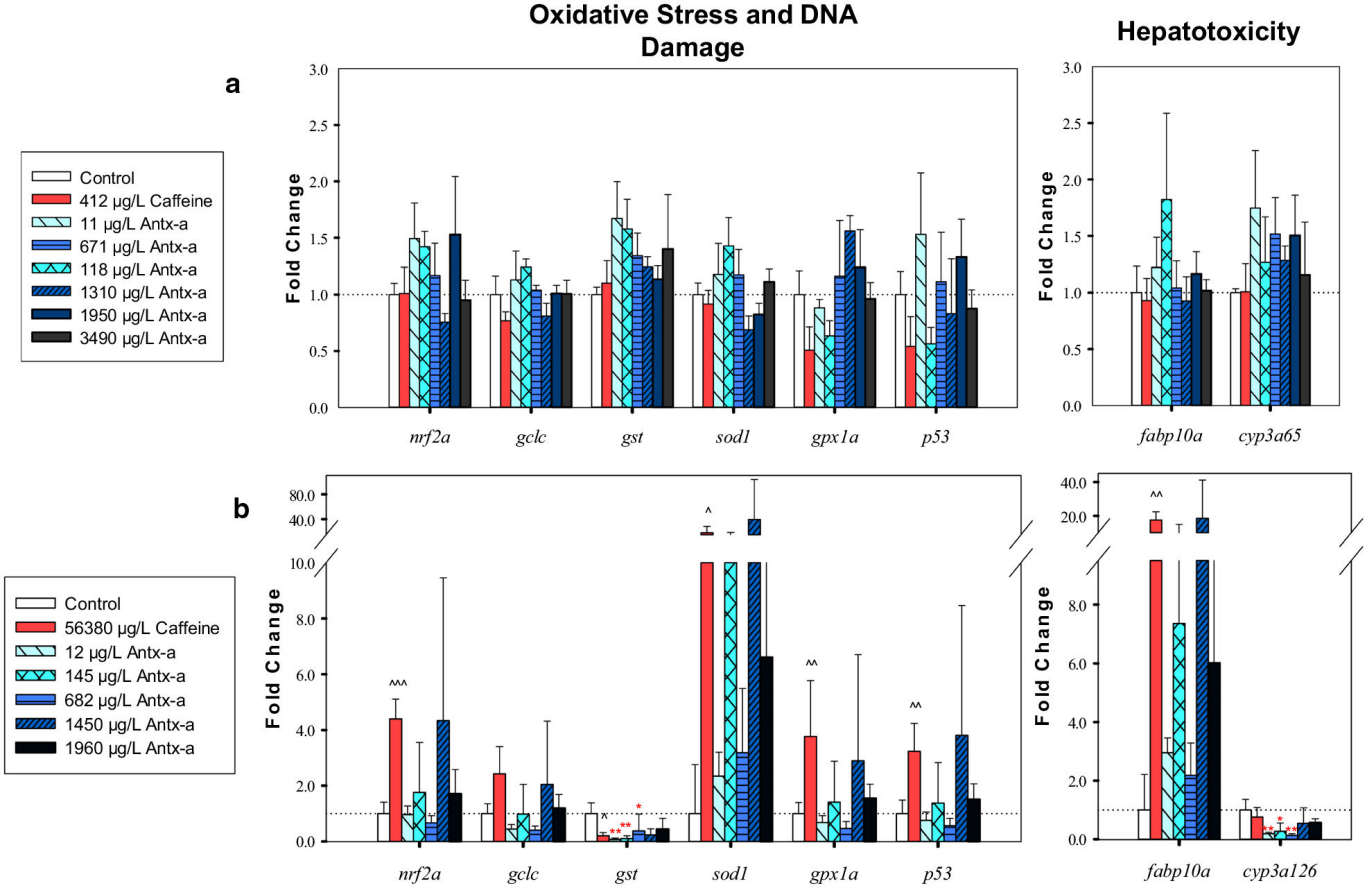
RT-qPCR oxidative stress, DNA damage, and hepatotoxicity gene expression data comparing larval fathead minnows exposed for 96 h to (±) antx-a or caffeine compared to the negative control. For zebrafish (**a**) or fathead minnows (**b**) exposed for 96 h to (±) antx-a or caffeine compared to the negative control. Transcript levels were normalized to housekeeping gene, *elongation factor 1 alpha* in zebrafish and *18s ribosomal RNA* in fathead minnow, using the 2^−ΔΔ*C*^_T_ method. In the (±) antx-a treatments, ANOVA and Dunnett’s post hoc were used to analyze treatment level responses compared to the negative control (*p < 0.05; **p < 0.01; ***p < 0.001). *T* tests were used to identify potential caffeine influences compared to the negative control (^ *p* < 0.05; ^^ *p* < 0.01; ^^^ *p* < 0.001), error bars (± SD). Zebrafish included 4 replicates with 21–24 larvae used for each treatment level. Fathead minnows included 4 replicates with 13–15 larvae used for each treatment level

In zebrafish, 412 μg/L caffeine significantly decreased (p < 0.05) the transcription of two genes related to central nervous system development: *ELAV like RNA binding protein 3* (*elavl3*) by twofold and *tubulin alpha 1* (*tuba1*) by 1.8-fold (Fig. 5a). Compared to the negative control, there was no significant difference in (±) antx-a-exposed zebrafish (α = 0.05) for any of the selected genes related to neurotoxicity, oxidative stress, DNA damage, or hepatotoxicity (Figs. 5a, 6a). In contrast, caffeine exposure in fathead minnows led to significant (*p* < 0.05) transcriptional increases in 5 of the 7 neurotoxicity-related genes (8–24-fold) (Fig. 5b), 5 of 6 oxidative stress and DNA damage-related genes (2–18-fold) (Fig. 6b), whereas *glutathione s-transferase* (*gst*) was significantly down regulated (5-fold) (Fig. 6b). In (±) antx-a-exposed fathead minnows, a trend towards transcriptional upregulation in most target genes was observed at the 1450 μg/L treatment level, with a notable 40-fold upregulation observed in *superoxide dismutase* (*sod1*). However, only *elavl3* was significantly changed (16-fold, *p* < 0 0.05). In the three lowest treatment levels (12–682 μg/L), gst (3–14-fold) and *cytochrome P450 Family 3 Subfamily A Member 126* (*cyp3a126*) (4–8-fold) were significantly downregulated (*p* < 0.05).

## Discussion

Antx-a is an emerging water quality threat [14] that has elicited spontaneous muscle spasms [48] and seizures [49] in mammals, but corresponding studies in alternative vertebrate models and other aquatic and terrestrial organisms are limited. In the present study, we hypothesized that (±) antx-a could cause similar responses in fish models, resulting in stimulatory behavior and increased changes in movement direction following waterborne exposure. Whereas zebrafish behavior was slightly stimulated, and thus, appears in general agreement with previous information from mammals, significantly less locomotion was observed in the fathead minnow, especially under dark conditions. However, photomotor response was not significantly affected in either model at the environmentally relevant concentrations of antx-a examined here. These contrasting responses may indicate different sites of action or receptor subtypes being activated by (±) antx-a. For example, nicotine differentially influences behavior in mammalian models, leading to either hyper- or hypolocomotor activity, depending on the site of action and which acetylcholine receptor subtype is activated [50]. Further mechanistic study of molecular initiation event(s) for antx-a is needed to understand sublethal influences on fish behavior.

Previous antx-a research has demonstrated largely decreased locomotor and other behaviors in various terrestrial organisms and *Daphnia* (Table 1). Rats and mice exposed to (+) antx-a (10–225 μg/kg), (±) antx-a (200–950 μg/kg), or an unspecified enantiomeric mixture (100–250 μg/kg), had lowered locomotor activity and operant responding (nicotine discrimination and food response) in behavioral assays compared to saline controls [51-54]. Higher doses (1,250,000–2,500,000 μg/kg) led to immediate extreme seizures, tachycardia, gasping, twitching, and coma before death [49]. In addition, antx-a decreased locomotion and other behaviors of roundworms in a dose- and time-dependent manner at 0.1–100 μg/kg antx-a, though here again enantiomers were not reported [55]. *Daphnia* locomotion was also lowered by (±) antx-a as they were immobilized with an EC_50_ of 2090 μg/L at 24 h and 1700 μg/L at 48 h [56]. (+) Antx-a altered swimming speed and limb activity in *Daphnia* within 10 s at 50,000 μg/L with decreased swimming speed at 24 h [59]. Antx-a exposure also altered *Daphnia* heart rate, thoracic limb activity and post-abdominal claw movement typically lowering these activities dependent on dose [57]. Decreased locomotion, particularly at higher speeds, following antx-a is consistent with the fathead minnows’ behavioral responses in the current study; however, this behavioral response profile was opposite from our observations with zebrafish, which were more active at higher speeds. Neuronal nicotinic acetylcholine receptors are highly conserved in vertebrates [58] with 17 nicotinic acetylcholine receptor subunits while invertebrates are less clear, though it has been suggested that *Drosophila* have 10 while *C. elegans* may have from 27 to 42 subunits [59]. Interestingly mammals have 16 genes encoding nicotinic acetylcholine receptors while zebrafish have 27 [60]. Understanding the diversity of the functions and subunit diversity of this receptor as it relates to antx-a toxicity may help elucidate why locomotor behaviors differ among species, including the current observations with zebrafish.

Fish behavioral studies with antx-a have indicated varied responses, though these efforts have examined different developmental stages, and studied various routes of exposure, concentrations, and sex-specific responses, which collectively challenge among experiment comparisons (summarized in Table 1). Zebrafish were exposed for 96 h starting at 4–6 h post-fertilization in the present study, but age-specific susceptibility to antx-a may exist and lead to different responses or thresholds for the endpoints examined here. For example, antx-a of an unspecified enantiomeric mixture at 400 μg/L altered zebrafish heart rate, decreasing 9% at 55 h and increasing 12% at 80 h [31]. One year old zebrafish exposed to 800 μg/kg (±) antx-a via intraperitoneal injection resulted in immediate rapid respiration, either frenetic swimming or complete lack of swimming with some moving backward, abnormal body position, and gulping for air [61]. Interestingly, this study also showed sex-specific proteomic responses [61] though it is unclear whether gender differences in adult fish exist for behavior. Rainbow trout immersed in an unspecified enantiomeric mixture of 129–499 μg/L antx-a led to multiple abnormal behaviors after 5 min including irregular/erratic swimming, jaw spasms, air gulping, and difficulty in maintaining equilibrium, though these fish largely recovered by 3 h [32]. Japanese medaka fish exposed to (±) antx-a through oral gavage from 200 to 20,000 μg/kg showed immediate neurotoxic effects including altered opercular movement, abnormal swimming, and muscle rigidity [62]. Since antx-a producing cultures of cyanobacteria may contain other biologically active molecules, studies examining behavioral responses to cyanobacteria were not included in Table 1, but remain necessary to understand behavioral toxicity of antx-a-producing cyanobacterial blooms in aquatic systems [15, 16, 21, 30, 63-65]. It is also important to note that much of the antx-a behavioral data with fish and other organisms (Table 1) did not employ the quantitative behavioral tracking software employed during the present study. Quantitative behavioral acquisition presents opportunities for robust and reproducible analyses in aquatic toxicology, particularly as behavioral responses are increasingly integrated within environmental protection efforts.

Early exposure to chemicals that alter neurotransmission, such as nicotine and chlorpyrifos, can lead to neurodevelopmental damage and abnormalities from inappropriate timing and intensity of neurotrophic actions [66, 67]. The neurodevelopmental linked genes examined here, *α1-tubulin* (*tuba1*), *ELAV like neuron-specific RNA binding protein 3* (*elavl3*), glia*l fibrillary acidic protein* (*gfap*), *myelin basic protein* (*mbp*), *neurogenin1* (*neurog1*), *sonic hedgehog a* (*shha*), and *synapsin IIa* (*syn2a*), have been shown to be transcribed in the first few days of fish development in neuronal stem cells, developing neurons, astrocytes, or oligodendrocytes, and are potential markers for rapid developmental neurotoxicity screening [68]. However, (±) antx-a had little effect on the transcription of zebrafish genes relating to neurotoxicity, which is consistent with no significant behavioral changes in this fish model, nor oxidative stress, DNA damage, and hepatotoxicity at the environmentally relevant treatment levels examined in the present study. Fathead minnow responses were more variable, though only 1 of 7 neurotoxicity-related genes, *elavl3,* was significantly transcriptionally altered. At the 1450 μg/L (±) antx-a treatment level, *elavl3*, which is involved in post-transcriptional regulation of neuronal RNA [69], was significantly upregulated in fathead minnows; this may be due to neurogenesis-related compensatory mechanisms. Similar compensatory regulation may be occurring for other upregulated genes at this concentration, though many showed lessened upregulation at the higher 1960 μg/L level. Upregulation of *elavl3* in developing zebrafish after exposure to tri-n-butyl phosphate, an organophosphate pesticide, was linked to significantly lowered fish relative free swimming speed [70]. However, other studies with the pesticide fenvalerate have reported decreased zebrafish swimming activity accompanied by downregulation of *elavl3* and other neurogenesis-linked genes [71]. Future studies with antx-a in these fish models should examine transcriptomic responses not included this analysis.

Oxidative stress can be linked to neurotoxicity in contributing to neuronal death [72] and neurobehavioral toxicity due to inhibition of antioxidant scavenging [73]. While no change in transcription was observed in zebrafish, *nuclear factor (erythroid-derived 2)-like 2a* (*nrf2a*), an endogenous sensor for cellular oxidative stress, was upregulated at the two highest levels of antx-a exposure in the fathead minnow. The function of *nrf2a* is highly evolutionarily conserved and works through antioxidant defense regulation [74]. *nrf2a* binds to antioxidant response element sequences, which results in the activation of antioxidant genes [74-76]. This likely accounts for the antioxidant genes in the current study following similar gene expression patterns because *nrf2a, gclc, gpx1a,* and *sod1* were upregulated at higher (±) antx-a treatment levels (1450–1960 μg/L). Previous research with cellular extracts containing antx-a and a purified toxin of an unknown enantiomer mixture has reported oxidative stress responses in multiple organisms and cell lines [77-80]. Both *gst* and *cytochrome P450 family 3 subfamily A polypeptide 126* (*cyp3a126*) transcription were significantly lowered in fathead minnows following (±) antx-a exposure, which also decreased swimming behavior at > 20 mm/s. Multiple studies have reported transcriptional changes in these genes associated with behavioral effects. Similarly to this study, bifenthrin, an insecticide, led to downregulated *cyp3a* and *gst* after 24 h exposure in fathead minnows [81] at the same treatment level (0.14 μg/L) that significantly decreased fathead minnow swimming performance in an earlier experiment [82]. This observation could possibly help link behavioral and gene transcription responses, but further study is needed. Lack of oxidative stress-related transcriptional responses in zebrafish in the current study could have resulted from treatment levels being too low to elicit responses, the exposure being too short (96 h), and/or the age difference between zebrafish and fathead minnows when experiments were initiated, among the other factors.

Fish are routinely employed during environmental quality efforts and are increasingly employed as alternative vertebrates during biomedical studies. Though zebrafish and fathead minnows represent two of the most common fish models, experiments examining sublethal toxicity of chemicals with both species are limited, particularly when molecular and behavioral endpoints are considered. In the present study, we observed fathead minnows to be more sensitive to (±) antx-a than zebrafish at the environmentally relevant concentrations examined. Other studies have demonstrated these common model organisms to have varying sensitivities to bisphenol A, cumene hydroperoxide, tert-butyl hydroperoxide [33], 1-heptanol, citalopram [34], 3-bromo-1-propanol, tris(2,3-dibromopropyl) phosphate [47], and caffeine [35], for which the fathead minnow model was 2–8 times more acutely sensitive than zebrafish. However, perfluorooctanoic acid [33] and sodium decyl sulfate [47] were 2–16 times more acutely toxic to zebrafish than fathead minnows. Further, chemicals can elicit opposite behavioral responses in both species, as illustrated by 3-chloro-1,2-propanediol and tris(2,3-dibromopropyl) phosphate, which both generally produced stimulatory effects in fatheads and refractory responses in zebrafish [47]. Advancing an understanding of the toxicokinetics and toxicodynamics (TKTD) of antx-a in these models will be important to define such among species differences. Unfortunately, very little research has been done on species-specific TKTD with antx-a.

Zebrafish embryos are relatively insensitive to many neurotoxic compounds, specifically those with molecular initiation events such as acetylcholinesterase inhibition, blockage of voltage-gated sodium channels, or interference with GABA-gated chlorine channels, compared to later life stages [83-85]. Though fathead minnow embryos have been shown to have lessened sensitivity to some neurotoxicants (e.g., fluoride, cadmium) [86], more research is needed to determine the extent to which interspecies insensitivities may exist for a wider range of neurotoxicants and neurotoxins. Age can also affect behavioral responses in larval fish, even in zebrafish born 3 days apart [42]. In the present study, we employed standard experimental designs from the OECD and the US EPA for zebrafish and fathead minnows, respectively. Subsequently, age of these fish models differed when experiments were initiated, and thus, may have contributed to the differential sensitivities observed here. FET tests for fathead minnows have been proposed that use embryos at similar ages to zebrafish in OECD FET studies [87, 88], yet this previous work focused on standard survival and growth response variables. Clearly, comparative toxicology research must be advanced to understand such interspecies differences and translate sublethal information among common model organisms employed for ecological and biomedical research.

## Conclusion

Though cyanobacteria blooms and other HABs appear to be increasing in magnitude, frequency and duration at the global scale, it remains uncommon among regulatory and resource management organizations to attribute degradation of inland surface water quality to HAB events [1]. Because comparative toxicology information for cyanotoxins, including antx-a, among vertebrates is lacking, in the present study we examined environmentally relevant levels of (±) antx-a and observed differential influences on swimming behavior and gene transcription in two common larval fish models. Importantly, we observed (±) antx-a to elicit opposite movement patterns in two common fish models, and further identified the fathead minnow model to be more sensitive to the toxin than zebrafish for behavioral and gene expression endpoints. Future studies are needed to understand these interspecies differences, influences of routes of exposure, the enantioselective toxicity of this compound, transcriptomic and proteomic responses, and to develop adverse outcome pathway(s) for this emerging water quality threat. Further, research is needed to determine whether antx-a predominately influences water quality risks during bloom events that may produce multiple known toxins and other biologically active molecules.

## Availability of data and materials

The datasets generated and/or analyzed during the current study are available from the corresponding author on reasonable request.

## Supplementary Material

Table SI1 **Additional file 1: Table SI.** List of genes and associated primer sequences used in this study for gene transcription analysis focusing on neurotoxicity, oxidative stress, DNA damage, and hepatotoxicity. In the table, ZF refers to zebrafish specific sequences, while FHM refers to fathead minnow specific sequences. Gene sequences that were not used from literature were designed for this study using the NCBI Primer-BLAST tool. *Efficiencies were determined using a standard curve of Ct values acquired from a 4-fold dilution series of cDNA (1, 1:4, 1:16) in duplicate.

## Figures and Tables

**Table 1:** Behavioral effects of the anatoxin-a toxin in various model systems. Only behavioral data from studies using the individual synthetic antx-a or extracted antx-a from culture were used. This excludes data from organisms exposed to antx-a producing cyanobacterial cells. Missing data were denoted with NA (not available)

Enantiomer	Toxin purity	Purified toxin extracted from culture?	Organism	Age	Treatment levels	Analytically verified?	Exposure method	Exposure duration	Study duration	Behavior type	Response	References
(+)	NA	No	Non-nicotine-tolerant male hooded rats	NA	10–200 μg/kg	NA	Subcutaneous injection	NA	60 min immediately after dosing	Locomotion	Rats showed decreased cage crosses (movement from one infrared beam to another across the cage) and repeated moves (successive interruptions of the same beam of light) compared to saline controls at 100 and 200 μg/kg (+) antx-a	Stolerman IP, Albuquerque EX, Garcha HS (1992) Behavioural effects of anatoxin, a potent nicotinic agonist, in rats. Neuropharmacology 31:311–314. https://doi.org/10.1016/0028-3908(92)90182-O
(+)	NA	No	Nicotine-tolerant male hooded rats	NA	10–200 μg/kg	NA	Subcutaneous injection	NA	60 min immediately after dosing	Locomotion	Rats showed decreased repeated moves and a tendency toward a reduced number of cage crosses at 200 μg/kg (+) antx-a from saline control	Stolerman IP, Albuquerque EX, Garcha HS (1992) Behavioural effects of anatoxin, a potent nicotinic agonist, in rats. Neuropharmacology 31:311–314. https://doi.org/10.1016/0028-3908(92)90182-O
(+)	NA	No	Male hooded rats trained to discriminate nicotine from saline	NA	10–200 μg/kg	NA	Subcutaneous injection	NA	60 min immediately after dosing	Nicotine discrimination stimulus	Rats showed decreased rates of operant responding in nicotine discrimination procedures and showed partially nicotine-like discriminative stimulus effects at 100 μg/kg (+) antx-a compared to saline controls	Stolerman IP, Albuquerque EX, Garcha HS (1992) Behavioural effects of anatoxin, a potent nicotinic agonist, in rats. Neuropharmacology 31:311–314. https://doi.org/10.1016/0028-3908(92)90182-O
(+)	NA	No	Male CD-1 mice	NA	30–50 μg/kg	Yes	Slow intravenous injection	15 min	> 1 min	Motor coordination	(+) Antx-a-treated mice showed clinical signs of cholinergic stimulation and CNS effects before death. 2 of 6 exposed to 50 μg/kg and 1 of 6 exposed to 30 μg/kg died. Surviving mice recovered and rota-rod testing was comparable to control	Fawell JK, Mitchell RE, Hill RE, Everett DJ (1999) The toxicity of cyanobacterial toxins in the mouse; II anatoxin-a. Hum Exp Toxicol 18:168–173. https://doi.org/10.1177/096032719901800306
Unknown	NA	No	Zebrafish (*Danio rerio*)	55 h	400 μg/L	NA	Immersion	NA	NA	Heart rate	Fish heart rate decreased 9% temporarily in antx-a treatment compared to control	Oberemm A, Becker J, Codd GA, Steinberg C (1999) Effects of cyanobacterial toxins and aqueous crude extracts of cyanobacteria on the development of fish and amphibians. Environ Toxicol 14:77–88. https://doi.org/10.1002/(SICI)1522-7278(199902)14:1<77::AIDTOX11>3.0.CO;2-F
Unknown	NA	No	Zebrafish (*Danio rerio*)	80 h	400 μg/L	NA	Immersion	NA	NA	Heart rate	Fish heart rate increased 12% temporarily in antx-a treatment compared to control	Oberemm A, Becker J, Codd GA, Steinberg C (1999) Effects of cyanobacterial toxins and aqueous crude extracts of cyanobacteria on the development of fish and amphibians. Environ Toxicol 14:77–88. https://doi.org/10.1002/(SICI)1522-7278(199902)14:<77::AIDTOX11>;3.0.CO;2-F
Unknown	≥ 90%	No	CD-1 mice	Adult	100–250 μg/kg	NA	Intraperitoneal injection	NA	5–10 min	Abnormal behavior	Decreased motor activity, altered gait, difficulty breathing, and convulsions in antx-a treatment mice	Rogers EH, Hunter ES, Moser VC, Phillips PM, Herkovits J, Muñoz L, Hall LL, Chernoff N (2005) Potential developmental toxicity of anatoxin-a, a cyanobacterial toxin. J Appl Toxicol 25:527–534. https://doi.org/10.1002/jat.1091
Unknown	≥ 90%	No	CD-1 mice	Pre-weaning	125–200 μg/kg	NA	In utero	Exposure from mother (intraperitoneal injection)	30–60 s	Neurological tests	No antx-a-related changes to righting reflex, negative geotaxis time, nor hang time	Rogers EH, Hunter ES, Moser VC, Phillips PM, Herkovits J, Muñoz L, Hall LL, Chernoff N (2005) Potential developmental toxicity of anatoxin-a, a cyanobacterial toxin. J Appl Toxicol 25:527–534. https://doi.org/10.1002/jat.1091
(+)	NA	No	Male Long Evans rats	Adult	75–225 μg/kg	NA	Injection	NA	30 min	Locomotion	(+) Antx-a dose dependent decreased horizontal and vertical activity, no tolerance was developed over weeks	MacPhail RC, Farmer JD, Jarema KA (2007) Effects of acute and weekly episodic exposures to anatoxin-a on the motor activity of rats: Comparison with nicotine. Toxicology 234:83–89. https://doi.org/10.1016/j.tox.2007.02.001
(±)	NA	No	Male Long Evans rats	Adult	200–950 μg/kg	NA	Injection	NA	30 min	Locomotion	(±) Antx-a dose dependent decreased horizontal and vertical activity at higher doses, no tolerance was developed over weeks	MacPhail RC, Farmer JD, Jarema KA (2007) Effects of acute and weekly episodic exposures to anatoxin-a on the motor activity of rats: Comparison with nicotine. Toxicology 234:83–89. https://doi.org/10.1016/j.tox.2007.02.001
(+)	NA	No	Male Long Evans rats	3 month	50–200 μg/kg	NA	Subcutaneous injection	5 min	Variable	Operant performance	Rats were trained to respond under a multiple variable ratio 30-response variable-interval 60 s schedule of food reinforcement. (+) Antx-a-exposed rats initially decreased in response and reinforcement rate. Though some tolerance occurred over 4 weeks of injections	Jarema KA, Poling A, MacPhail RC (2008) Effects of weekly exposure to anatoxin-a and nicotine on operant performance of rats. Neurotoxicol Teratol 30:220–227. https://doi.org/10.1016/j.ntt.2008.02.001
Unknown	98%	No	Rainbow trout (*Oncorhynchus mykiss*)	3 month	129–499 μg/L	Yes	Immersion	96 h	5 min–3 h	Abnormal behavior	In all antx-a treatments fish showed irregular/erratic swimming, jaw spasms, air gulping at surface, difficulty in maintaining equilibrium after 5 min with fish recovering by 3 h	Osswald J, Azevedo J, Vasconcelos V, Guilhermino L (2011) Experimental determination of the bioconcentration factors for anatoxin-a in juvenile rainbow trout (*Oncorhynchus mykiss*). Proc Int Acad Ecol Environ Sci 1:77–86
(±)	NA	No	Cladocera (*Daphnia magna*)	NA	> 4000 μg/L	NA	Immersion	24 h, 48 h	NA	Free swimming	24 h EC50 was 2090 μg/L (±) antx-a and 48 h EC50 was 1700 μg/L (±) antx-a daphnia were unable to swim freely	Sierosławska A (2013) Evaluation of the Sensitivity of Organisms Used in Commercially Available Toxkits to Selected Cyanotoxins. Pol J Environ Stud 22:1817–1823
(±)	NA	No	Rotifer (*Brachionus calyciflorus*)	NA	> 4000 μg/L	NA	Immersion	24 h	NA	Free swimming	24 h EC50 was > 4000 μg/L (±) antx-a rotifers were unable to swim freely	Sierosławska A (2013) Evaluation of the Sensitivity of Organisms Used in Commercially Available Toxkits to Selected Cyanotoxins. Pol J Environ Stud 22:1817–1823
(±)	NA	No	Male Wistar strain albino rats	5–7 weeks	1250–2500 mg/kg, 1,250,000–2,500,000 μg/kg	NA	Subcutaneous injection	Variable	Variable	Abnormal behavior	Extreme seizures, tremors, tachycardia, gasping, fasciculation, acute asphyxiation, latency followed up by twitching, decrease in locomotor activities, coma, before death	Banerjee S, Chattopadhyay P, Ghosh A, Pathak MP, Gogoi J, Veer V (2014) Protection by a transdermal patch containing eserine and pralidoxime chloride for prophylaxis against (±)-Anatoxin A poisoning in rats. Eur J Pharm Sci 56:28–36. https://doi.org/10.1016/j.ejps.2014.01.013
Unknown	NA	No	Wild-type roundworms strain N2 (*Caenorhabditis elegans*)	L4 larvae	.1–100 μg/L	NA	Added to agar	24 h or 72 h	20 s	Locomotion	Antx-a exposure led to dose dependent decreased body bend frequency at 24-h and 72-h exposure and lowered move length at all concentrations in both 24-h and 72-h exposure	Ju J, Saul N, Kochan C, Putschew A, Pu Y, Yin L, Steinberg C (2014) Cyanobacterial Xenobiotics as Evaluated by a Caenorhabditis elegans Neurotoxicity Screening Test. Int J Environ Res Public Health 11:4589–4606. https://doi.org/10.3390/ijerph110504589
Unknown	NA	No	Wild-type roundworms strain N2 (*Caenorhabditis elegans*)	L4 larvae	.1–100 μg/L	NA	Added to agar	24 h or 72 h	3 times over 60 s	Food intake	Decreased pharyngeal pumping 10–100 μg/L antx-a in 24-h exposed worms and 1–100 μg/L antx-a in 72-h exposed worms	Ju J, Saul N, Kochan C, Putschew A, Pu Y, Yin L, Steinberg C (2014) Cyanobacterial Xenobiotics as Evaluated by a Caenorhabditis elegans Neurotoxicity Screening Test. Int J Environ Res Public Health 11:4589–4606. https://doi.org/10.3390/ijerph110504589
Unknown	NA	No	Wild-type roundworms strain N2 (*Caenorhabditis elegans*)	L4 larvae	.1–100 μg/L	NA	Added to agar	24 h or 72 h	50 s	Defecation assay	Lowered defecation period interval at 100 μg/L antx-a in 2-h exposed worms	Ju J, Saul N, Kochan C, Putschew A, Pu Y, Yin L, Steinberg C (2014) Cyanobacterial Xenobiotics as Evaluated by a Caenorhabditis elegans Neurotoxicity Screening Test. Int J Environ Res Public Health 11:4589–4606. https://doi.org/10.3390/ijerph110504589
Unknown	NA	No	Wild-type roundworms strain N2 (*Caenorhabditis elegans*)	L4 larvae	.1–100 μg/L	NA	Added to agar	24 h or 72 h	1 h	Chemotaxis (NaCl)	Lowered chemical index .1–100 μg/L antx-a-exposed worms after 24- and 72-h exposure	Ju J, Saul N, Kochan C, Putschew A, Pu Y, Yin L, Steinberg C (2014) Cyanobacterial Xenobiotics as Evaluated by a Caenorhabditis elegans Neurotoxicity Screening Test. Int J Environ Res Public Health 11:4589–4606. https://doi.org/10.3390/ijerph110504589
Unknown	NA	No	Wild-type roundworms strain N2 (*Caenorhabditis elegans*)	L4 larvae	.1–100 μg/L	NA	Added to agar	24 h or 72 h	1 h	Thermotaxis	Lowered fraction of worms in 20 C category for 1–100 μg/L antx-a after 24-h exposure and lowered fraction of worms in 20 C and movement between 20 and 25C category for .1–100 μg/L antx-a-exposed worms for 72 h	Ju J, Saul N, Kochan C, Putschew A, Pu Y, Yin L, Steinberg C (2014) Cyanobacterial Xenobiotics as Evaluated by a Caenorhabditis elegans Neurotoxicity Screening Test. Int J Environ Res Public Health 11:4589–4606. https://doi.org/10.3390/ijerph110504589
Unknown	NA	No	Wild-type roundworms strain N2 (*Caenorhabditis elegans*)	L4 larvae	.1–100 μg/L	NA	Added to agar	24 h or 72 h	1 h	Mechanical sensory stimulus	No nose touch response change from control for any antx-a concentration or exposure duration	Ju J, Saul N, Kochan C, Putschew A, Pu Y, Yin L, Steinberg C (2014) Cyanobacterial Xenobiotics as Evaluated by a Caenorhabditis elegans Neurotoxicity Screening Test. Int J Environ Res Public Health 11:4589–4606. https://doi.org/10.3390/ijerph110504589
(±)	98%	No	Zebrafish (*Danio rerio*)	1 year	800 μg/kg	NA	I.p. injection	Immediate observation	After 5 min	Abnormal behavior	(±) Antx-a-exposed fish showed rapid respiration as evidenced by opercular movement, frenetic swimming or complete lack of swimming with some moving backward, abnormal body position, gulping for air	Carneiro M, Gutiérrez-Praena D, Osório H, Vasconcelos V, Carvalho AP, Campos A (2015) Proteomic analysis of anatoxin-a acute toxicity in zebrafish reveals gender specific responses and additional mechanisms of cell stress. Ecotoxicol Environ Saf 120:93–101. https://doi.org/10.1016/j.ecoenv.2015.05.031
(+)	≥ 98%	Dolichospermum flos-aquae (prev. Anabaena flos-aquae)	Cladocera (*Daphnia magna*)	Neonate	500–50,000 μg/L	NA	Immersion	10 s, 5 min, 15 min, 30 min, 2 h, 24 h	≥ 1 min	Swimming speed	500, 2500, 50,000 μg/L (+) antx-a-treated Daphnia showed some increased movement before 24 h, while all (+) antx-a concentrations showed roughly 5 times lowered swimming speed at 24 h compared to controls	Bownik A, Pawlik-Skowrońska B (2019) Early indicators of behavioral and physiological disturbances in Daphnia magna (Cladocera) induced by cyanobacterial neurotoxin anatoxin-a. Sci Total Environ 695:133,913. https://doi.org/10.1016/j.scitotenv.2019.133913
(+)	≥ 98%	Dolichospermum flos-aquae (prev. Anabaena flos-aquae)	Cladocera (*Daphnia magna*)	Neonate	500–50,000 μg/L	NA	Immersion	10 s, 5 min, 15 min, 30 min, 2 h, 24 h	≥ 1 min	Abnormal circular movements	2500–50,000 μg/L (+) antx-a-treated Daphnia showed increased circular movements from 10 s to 30 min of exposure, though all concentrations were similar to control at 24 h	Bownik A, Pawlik-Skowrońska B (2019) Early indicators of behavioral and physiological disturbances in Daphnia magna (Cladocera) induced by cyanobacterial neurotoxin anatoxin-a. Sci Total Environ 695:133,913. https://doi.org/10.1016/j.scitotenv.2019.133913
(+)	≥ 98%	Dolichospermum flos-aquae (prev. Anabaena flos-aquae)	Cladocera (*Daphnia magna*)	Neonate	500–50,000 μg/L	NA	Immersion	2 h or 24 h	≥ 1 min	Heart rate	While 500 and 2500 μg/L (+) antx-a-treated Daphnia showed slightly lowered heart rate compared to control, 10,000 and 50,000 μg/L treated Daphnia showed highly decreased heart rate. All exposed Daphnia showed time-dependent decreases between 2- and 24-h exposure	Bownik A, Pawlik-Skowrońska B (2019) Early indicators of behavioral and physiological disturbances in Daphnia magna (Cladocera) induced by cyanobacterial neurotoxin anatoxin-a. Sci Total Environ 695:133,913. https://doi.org/10.1016/j.scitotenv.2019.133913
(+)	≥ 98%	Dolichospermum flos-aquae (prev. Anabaena flos-aquae)	Cladocera (*Daphnia magna*)	Neonate	500–50,000 μg/L	NA	Immersion	2 h or 24 h	≥ 1 min	Thoracic limb activity	500 μg/L (+) antx-a treated Daphnia showed slightly higher thoracic limb activity at 2 h while 2500– 50,000 μg/L (+) antx-a treated Daphnia showed lowered limb activity with 50,000 μg/L (+) antx-a leading to 0 beats per minute at 2 h and 10,000 μg/L leading to 0 beats per minute after 24 h	Bownik A, Pawlik-Skowrońska B (2019) Early indicators of behavioral and physiological disturbances in Daphnia magna (Cladocera) induced by cyanobacterial neurotoxin anatoxin-a. Sci Total Environ 695:133,913. https://doi.org/10.1016/j.scitotenv.2019.133913
(+)	≥ 98%	Dolichospermum flos-aquae (prev. Anabaena flos-aquae)	Cladocera (*Daphnia magna*)	Neonate	500–50,000 μg/L	NA	Immersion	2 h or 24 h	≥ 1 min	Postabdominal claw movement	500–2500 μg/L (+) antx-a treated Daphnia showed increased claw movement while 10,000– 50,000 μg/L (+) antx-a treated Daphnia showed no claw activity for either time point	Bownik A, Pawlik-Skowrońska B (2019) Early indicators of behavioral and physiological disturbances in Daphnia magna (Cladocera) induced by cyanobacterial neurotoxin anatoxin-a. Sci Total Environ 695:133,913. https://doi.org/10.1016/j.scitotenv.2019.133913
(±)	NA	No	Female Japanese medaka (*Oryzias latipes*)	> 6 month	200–20,000 μg/kg	Yes	Oral gavage		Immediate observation after dosing	Abnormal behavior	< 6670 μg/kg (±) antx-a no apparent symptoms of toxicosis, at 20,000 μg/kg (±) antx-a within 5 min of exposure stop or lowered opercular movement, abnormal swimming, muscle rigidity. All but one fish at 10,000 μg/kg (±) antx-a still breathing with cessation at 15 min	Colas S, Duval C, Marie B (2020) Toxicity, transfer and depuration of anatoxin-a (cyanobacterial neurotoxin) in medaka fish exposed by single-dose gavage. Aquat Toxicol 222:105,422. https://doi.org/10.1016/j.aquatox.2020.105422
(±)	> 98%	No	Zebrafish (*Danio rerio*)	Embryo 4–6 h post-fertilization	11–3490 μg/L	Yes	Immersion	96 h	50 min	Larval photomotor response/locomotion	Consistent larval photomotor response to control. Stimulatory trend in movement in 11–1950 μg/L (±) antx-a-exposed fish showing more locomotion at highest speed (> 20 mm/s), then lowered movement at all speeds at 3490 μg/L (±) antx-a. Both findings more pronounced in light periods vs. dark	Current study
(±)	> 98%	No	Fathead minnow (*Pimephales promelas*)	Larvae < 48 h post-hatch	12–1960 μg/L	Yes	Immersion	96 h	50 min	Larval photomotor response/locomotion	Consistent larval photomotor response to control. Refractory movement in 145–1960 μg/L (±) antx-a-exposed fish showing less locomotion at highest speed (> 20 mm/s). Fairly consistent results in light and dark periods	Current study

## Abbreviations

Antx-a: Anatoxin-a
ANOVA: Analysis of variance
CAS: Chemical Abstracts Service
EPA: United States Environmental Protection Agency
FET: Fish embryo toxicity
hpf: Hours post-fertilization
ip: Intraperitoneal
LC–MS/MS: Liquid chromatography tandem mass spectrometry
OECD: Organisation for economic co-operation and development
RHW: Reconstituted hard water
RT-qPCR: Reverse transcription quantitative polymerase chain reaction
